# Eighty‐six cases of clinical characteristics and outcomes of systemic lupus erythematosus‐associated macrophage activation syndrome: A meta‐analysis study

**DOI:** 10.1002/iid3.1364

**Published:** 2024-08-07

**Authors:** Jingya Wang, Wei Rong, Haotian Yan

**Affiliations:** ^1^ Department of Rheumatology, Wuhu Hospital East China Normal University (The Second People's Hospital of Wuhu) Wuhu Anhui China

**Keywords:** disease characteristics, disease prognosis, MAS, SLE

## Abstract

**Objective:**

To improve our understanding of systemic lupus erythematosus (SLE)‐macrophage activation syndrome (MAS).

**Methods:**

A systematic review was performed, to retrieve all those papers on patients with SLE‐MAS, in individual or aggregated form. The data in each of these medical records were extracted and analyzed to identify the characteristics of SLE‐MAS.

**Results:**

A total of 86 SLE‐MAS patients were included (25 males and 61 females. The mean (±standard error of the mean) age was 31.21 ± 1.694 years. MAS occurred as the initial presentation of SLE in 47 people (54.65%) and during the course of SLE in 39 (45.35%). A coinfection was reported in 23 (26.74%) patients. The mean Lupus Erythematosus Disease Activity Index 2000 (SLEDAI‐2K) score was 16.54 ± 0.9462. Overall, 10 patients (11.63%) died. The SLEDAI‐2K score was higher in patients with MAS as an initial manifestation of SLE than in those where MAS occurred during the course of SLE. The proportion of patients receiving steroid pulse therapy was lower in patients with coinfections. The deceased group demonstrated lower platelet and ferritin levels. Multiple regression analysis revealed that age and thrombocytopenia were independent factors associated with poor prognosis. In receiver operating characteristic analysis, a platelet count cutoff value of ≤47 × 10^9^/L was a predictor of poor outcome.

**Conclusions:**

SLE‐MAS patients demonstrated high lupus activity, and lupus activity was especially higher in patients with MAS as an initial manifestation. Lupus activity was the predominant trigger of lupus MAS. Thrombocytopenia was an independent factor for poor prognosis.

## Highlights

- Lupus activity was especially high in patients with macrophage activation syndrome (MAS) as an initial manifestation.
- Lupus activity is the predominant trigger for lupus‐MAS.
- Thrombocytopenia is a predictor of poor prognosis in patients with systemic lupus erythematosus‐MAS.

## Graphical abstract

1. Lupus activity was especially high in patients with macrophage activation syndrome (MAS) as an initial manifestation. 2. Lupus activity is the predominant trigger for lupus‐MAS. 3. Thrombocytopenia is a predictor of poor prognosis in patients with systemic lupus erythematosus‐MAS.

## 1 INTRODUCTION

Hemophagocytic lymphohistiocytosis (HLH) is a life‐threatening and devastating disease^1^ that presents as a severe hyperinflammatory condition with clinical features such as fever, hepatosplenomegaly, cytopenia, and macrophage activation in hemopoietic organs. HLH can be divided into two categories: primary and secondary. HLH can occur secondary to rheumatic diseases as macrophage activation syndrome (MAS), with systemic lupus erythematosus (SLE‐MAS) accounting for more than half of all cases of MAS.^2^, ^3^ However, only a few studies have reported on the specific clinical features of SLE‐MAS. To date, research suggests that a high Lupus Erythematosus Disease Activity Index 2000 score^4^, ^5^ and cytopenia^4^ are the key characteristics of MAS in SLE patients. While reports of MAS as the initial manifestation of lupus are increasing,^6^, ^7^, ^8^ MAS can occur anytime during the course of lupus.^9^ In particular, MAS has been reported to develop in lupus in association with infections^9^, ^10^ or with a flare in SLE activity.^11^

Several important characteristics of SLE‐MAS remain to be determined. Differences in the MAS characteristics at the initial manifestation of SLE and those occurring during the course of SLE are not well known. While disease activity and associated infections are both known to trigger MAS, it is not clear which mechanism plays the predominant role in triggering SLE‐MAS. Finally, factors associated with SLE‐MAS prognosis are rarely be explored.

If we consider individual case reports as providing important (but fragmented) information about SLE‐MAS, we can synthesize inferences. Thus, we collated the records of 86 patients with SLE‐MAS based on published case reports. The data in each of these medical records were carefully extracted and statistically analyzed to identify the characteristics of SLE‐MAS. We also compared MAS characteristics that occurred at the initial manifestation of SLE to those that occurred during the course of SLE. Third, we compared the characteristics of MAS triggered by lupus disease activity or a coinfection. Finally, we propose the prognoses of these cases of SLE‐MAS. Our research findings should provide rheumatologists with a better understanding of this refractory and life‐threatening disorder.

## 2 PATIENTS AND METHODS

### 2.1 Search strategy and criteria for case selection

A systematic literature review was performed using the databases systematically: PubMed, Embase, Cochrane, Web of Science, Wanfang, SinoMed, CNKI, and Cqvip. The following medical subject headings were combined for the search: hemophagocytic syndrome, hemophagocytosis, hemophagocytic histiocytosis, or macrophage activation syndrome. These headings were combined with the subheadings “systemic lupus erythematosus,” or “SLE.” Articles were subsequently reviewed and included if the following criteria were met: (1) clinical features, laboratory findings, physical examination data, treatments, and the outcome of each case were clearly described, (2) meets SLE diagnostic criteria, (3) meets MAS diagnostic criteria, (4) MAS occurred during an active phase or at the onset of underlying SLE, and (5) patients showed no evidence of any other known underlying cause of hemophagocytic syndrome, such as drugs or malignancies. All relevant articles up until December 2020 were included. Two researchers worked independently. Any disagreements were resolved through discussion with a third reviewer.

SLE was defined according to the 1997 revised criteria for the classification of SLE, or the classification standard released by the Systemic Lupus International Collaborative Clinics (SLICC) in 2012,^12^ or the classification standard jointly released by the European League Against Rheumatology (EULAR)/ACR in 2019.^13^ MAS was diagnosed according to the criteria for HLH revised by the International Organization Cell Association in 2004,^14^ meeting five of the eight indicators. The complete screening process is described in Supporting Information S2: Table 1.

#### 2.1.1 Data analysis

A uniform evaluation chart was used to extract relevant information from each case. Data was extracted during the onset of MAS. Symptoms and signs included clinical signs of SLE (cutaneous involvement, oral ulcer, alopecia, neuropsychiatric SLE (NPSLE), vasculitis, arthritis, and nephritis), signs of MAS (fever, lymphadenopathy, hepatomegaly, and splenomegaly), laboratory findings of SLE and MAS (antinuclear antibody [ANA]), antidouble‐stranded DNA antibody (ds‐DNA), anti‐Smith antibody, anti‐Sjögren's syndrome‐related antigen A (anti‐SSA), anti‐Sjögren's syndrome‐related antigen B (anti‐SSB), complement C3 levels, complement C4 levels, anticardiolipin levels, direct Coombs test results, peripheral blood cell counts, ferritin levels, fibrinogen levels, triglyceride levels, and serum liver enzyme levels (lactate dehydrogenase [LDH]), C‐reactive protein (CRP), erythrocyte sedimentation rate (ESR), and outcomes (survival/mortality) for each case. SLE activity was evaluated in each lupus patient using SLEDAI‐2K.

#### 2.1.2 Statistical analysis

The data are presented as the mean ± standard error of the mean (SEM) unless otherwise specified. Univariate analysis was performed to compare two different groups using either the Student's unpaired *t* test or the *χ* ^2^ test, as appropriate. Univariate analysis of clinical and laboratory data was performed to identify prognostic variables. Multivariate logistic regression models were created using categorical predictors. Discrimination of the prediction model was evaluated using receiver operating characteristic (ROC) analysis. Univariate analysis was performed using GraphPad Prism (version 8.0.2), multivariate logistic regression was performed using SPSS (version 23.0) software, and ROC analysis was performed using MedCalc software. All tests were two‐tailed. We also reported hazard ratios (HRs) and 95% confidence intervals (95% CIs). A *p* value of .05 was used to test for statistical significance.

## 3 RESULTS

### 3.1 Characteristics of SLE‐MAS

**Figure 1:**
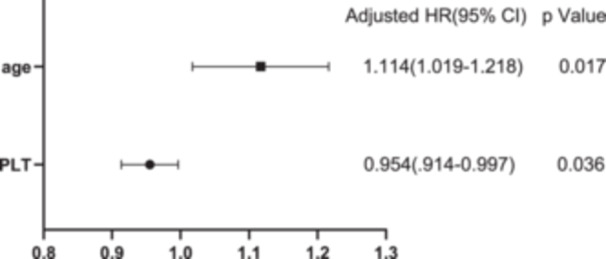
Multivariate logistic regression analysis for the predictors of poor prognosis. CI, confidence interval; HR, hazard ratio; PLT, platelet.

In total, 86 SLE‐MAS patients were included in our retrospective review (Table 1). The geographical distribution of the included patients is shown in Supporting Information S1: Figure 1. Overall, there were 25 men (29.07%) and 61 women, and a mean age (mean ± SEM) of 31.21 ± 1.694 years. The prevalence of symptoms and signs in the SLE patients was as follows: cutaneous involvement, 63.95%; nephritis, 51.16%; oral ulcer, 36.05%; arthritis, 34.88%; serositis, 26.74%; fever, 75.58%; splenomegaly, 46.51%; hepatomegaly, 40.7%; and lymphadenopathy, 34.88%. The mean ± SEM white blood cell (WBC) count, hemoglobin (Hgb) level, platelet count, LDH, ferritin, CRP, ESR, triglyceride, C3, C4, fibrinogen level, and SLEDAI‐2K score are shown in Table 1. The timing of SLE‐ MAS manifestation was as follows: MAS occurred as an initial presentation of SLE in 47 patients (54.65%), and 39 (45.35%) occurred during the course of SLE. MAS occurred with an infection (virus, bacteria, or fungi) in 23 patients (26.74%). The overall mortality rate was 11.63% (10 patients).

**Table 1:** Characteristics of SLE‐MAS.

	Total (*n* = 86)
Age, mean ± SEM (years)	31.21 ± 1.694
Male: female ratio (%)	25/61 (29.07)
MAS occurred as an initial presentation of SLE no. (%)	47/86 (54.65)
MAS occurred during the course of SLE no. (%)	39/86 (45.35)
Coinfected, no./total no. (%)	23/86 (26.74)
SLEDAI‐2K	16.54 ± 0.9462
Clinical	
Cutaneous involvement, no./total no. (%)	53/86 (63.95)
Nephritis, no./total no. (%)	44/86 (51.16)
Oral ulcer, no./total no. (%)	31/86 (36.05)
Arthritis, no./total no. (%)	30/86 (34.88)
Fever, no./total no. (%)	65/86 (75.58)
Splenomegaly, no./total no. (%)	40/86 (46.51)
Hepatomegaly, no./total no. (%)	35/86 (40.7)
Lymphadenopathy, no./total no. (%)	30/86 (34.88)
Hematological	
WBC count, mean ± SEM (range) × 10^9^/L	2.501 ± 0.2880
Hgb, mean ± SEM (range) g/L	89.91 ± 2.182
Platelet count, mean ± SEM (range) × 10^9^/L	83.20 ± 8.188
Serological	
LDH, mean ± SEM (range) IU/L	1392 ± 126.1
Triglyceride (mg/dL)	342.6 ± 28.29
Ferritin, mean ± SEM (range) ng/mL	36324 ± 20071
CRP, mean ± SEM (range) mg/L	62.67 ± 20.62
ESR (mm/H)	59.87 ± 4.369
Biochemical	
ANA positive, no./total no. (%)	79/86 (91.86)
Anti‐dsDNA antibody positive, no./total no. (%)	65/86 (75.56)
Anti‐Smith antibody positive, no./total no. (%)	20/86 (23.26)
Direct Coombs test positive, no./total no. (%)	13/86 (15.12)
Complement C3 (g/L)	0.4798 ± 0.03966
Complement C4 (g/L)	0.09932 ± 0.01123
Therapy and outcome	
Corticosteroids, no./total no. (%)	86/86 (100)
methylprednisolone pulse ≥ 500 mg, no./total no. (%)	73/86 (84.88)
Prednisolone ≥ 100 mg, no./total no. (%)	84/86 (97.67)
IVIG, no./total no. (%)	37/86 (43.02)
Cyclosporine, no./total no. (%)	25/86 (29.07)
Plasma exchange, no./total no. (%)	9/86 (10.47)
Patients deceased, no./total no. (%)	10/86 (11.63)

Abbreviations: ANAs, antinuclear antibodies; CRP, C‐reactive protein; ds‐DNA, antidouble‐stranded DNA; ESR, erythrocyte sedimentation rate; Hgb, hemoglobin; IVIG, intravenous immunoglobulin; LDH, lactate dehydrogenase; MAS, macrophage activation syndrome; SEM, standard error of the mean; SLE, systemic lupus erythematosus; SLEDAI‐2K, Lupus Erythematosus Disease Activity Index 2000; WBC, white blood cell.

### 3.2 Comparison between MAS at initial SLE manifestation and during the course of SLE

Clinical symptoms, clinical signs, treatments, and outcomes were compared between patients with MAS as an initial manifestation of SLE and patients in which MAS occurred during the course of lupus (Table 2). A significant difference was observed in the proportion of male patients in the two groups (*p* = .0109). Nineteen (40.43%) men had MAS as an initial presentation of SLE, and only six (15.38%) men had MAS during the course of lupus. Patients with MAS as an initial manifestation of SLE were more likely to present with an oral ulcer (46.81% vs. 23.08%, *p* = .0225), serositis (38.30% vs. 12.82%, *p* = .0079), and lymphadenopathy (48.94% vs. 17.95%, *p* = .0027). A greater proportion of patients with MAS as an initial manifestation of SLE had positive ANA results and were more likely to have a positive direct Coombs test than patients who had MAS during the course of lupus (97.87% vs. 84.62%, *p* = .0431; 23.40% vs. 5.13%, *p* = .0185). C3 levels (0.4174 ± 0.04605 vs. 0.5646 ± 0.06724, *p* = .0072) and C4 levels (0.08030 ± 0.01303 vs. 0.1255 ± 0.01930, *p* = .0321) were significantly lower in patients with MAS as an initial manifestation of SLE compared with patients with MAS during the course of lupus. SLEDAI‐2K scores (18.34 ± 1.240 vs. 14.45 ± 1.393, *p* = .03) were significantly higher in patients with MAS as an initial manifestation of SLE. Finally, patients with MAS during the course of lupus were more likely to be treated with cyclosporine (14.89% vs. 46.15%, *p* = .0015).

**Table 2:** Characteristics of MAS as an initial manifestation of SLE or during the course of lupus.

	MAS as an initial presentation of SLE	MAS in the course of lupus	*p*
	*n* = 47 (54.65%)	*n* = 39 (45.35%)	
Age (mean ± SEM [range] years)	32.77 ± 2.680	29.33 ± 1.873	.6991
Male: female ratio, no./total no. (%)	19/47 (40.43)	6/39 (15.38)	.0109
Coinfected, no./total no. (%)	12/47 (25.53.64)	11/39 (28.20)	.8120
SLEDAI‐2K	18.34 ± 1.240	14.45 ± 1.393	.03
Oral ulcer, no./total no. (%)	22/47 (46.81)	9/39 (23.08)	.0225
Serositis, no./total no. (%)	18/47 (38.30)	5/39 (12.82)	.0079
Nephritis, no./total no. (%)	26/47 (55.32)	18/39 (46.15)	.3973
Cutaneous involvement, no./total no. (%)	32/47 (68.09)	21/39 (53.85)	.1764
Vasculitis, no./total no. (%)	6/47 (12.77)	4/39 (10.26)	>.9999
Arthritis, no./total no. (%)	16/47 (34.04)	14/39 (35.90)	.8574
Fever, no./total no. (%)	35/47 (74.47)	30/39 (76.92)	.7919
Hepatomegaly, no./total no. (%)	19/47 (40.43)	16/39 (41.03)	.955
Splenomegaly, no./total no. (%)	18/47 (38.30)	22/39 (56.41)	.0936
Lymphadenopathy, no./total no. (%)	23/47 (48.94)	7/39 (17.95)	.0027
WBC count, mean ± SEM (range) × 10^9^/L	2.568 ± 0.4718	2.607 ± 0.2572	.0666
Hgb, mean ± SEM (range) g/L	87.91 ± 3.205	92.14 ± 2.926	.4798
Platelet count, mean ± SEM (range) × 10^9^/L	93.19 ± 13.81	71.32 ± 6.935	.5601
Direct Coombs test positive, no./total no. (%)	11/47 (23.40)	2/39 (5.13)	.0185
Complement C3 (g/L)	0.4174 ± 0.04605	0.5646 ± 0.06724	.0072
Complement C4 (g/L)	0.08030 ± 0.01303	0.1255 ± 0.01930	.0321
LDH, mean ± SEM (range) IU/L	1432 ± 199.4	1352 ± 156.8	.7548
Triglyceride (mg/dL)	361.5 ± 32.51	306.9 ± 54.28	.2236
Ferritin, mean ± SEM (range) ng/mL	58703 ± 35123	6953 ± 1832	.2881
CRP, mean ± SEM (range) mg/L	84.54 ± 45.10	45.17 ± 9.146	.3475
ESR (mm/H)	62.56 ± 7.077	56.96 ± 5.009	.8595
Therapy			
Steroid pulse therapy, no./total no. (%)	39/47 (82.98)	34/39 (87.18)	.5882
IVIG, no./total no. (%)	17/47 (36.17)	20/39 (51.28)	.1588
Cyclosporine, no./total no. (%)	7/47 (14.89)	18/39 (46.15)	.0015
IV CYC, no./total no. (%)	6/47 (12.77)	6/39 (15.38)	.7272
Supportive intensive care, no./total no. (%)	3/47 (63.83)	1/39 (2.56)	.6228
Patients deceased, no./total no. (%)	5/47 (10.64)	5/39 (12.82)	.7392

Abbreviations: CRP, C‐reactive protein; ESR, erythrocyte sedimentation rate; Hgb, hemoglobin; IVIG, intravenous immunoglobulin; IV CYC, intravenous cyclophosphamide; LDH, lactate dehydrogenase; MAS, macrophage activation syndrome; SEM, standard error of the mean; SLE, systemic lupus erythematosus; SLEDAI‐2000, Lupus Erythematosus Disease Activity Index 2000; WBC, white blood cell.

### 3.3 Comparison between SLE‐MAS with infection and SLE‐MAS without infection

A total of 23 (26.74%) patients presented with an infection (Supporting Information S2: Table 2). SLE and MAS symptoms and signs, therapies (other than anti‐infection therapies), and outcomes were compared in patients with and without infections (Supporting Information S2: Table 3). No significant differences in C3 levels, C4 levels, WBC counts, Hgb levels, platelet counts, CRP levels, ESR levels, SLEDAI‐2K scores, or patient outcomes were observed between the two groups. In those with a coinfection, the proportion of patients receiving steroid pulse therapy and having cutaneous involvement was lower, and the proportion of serositis involvement was higher compared to those without infections.

### 3.4 SLE‐MAS patient outcomes

**Figure 2:**
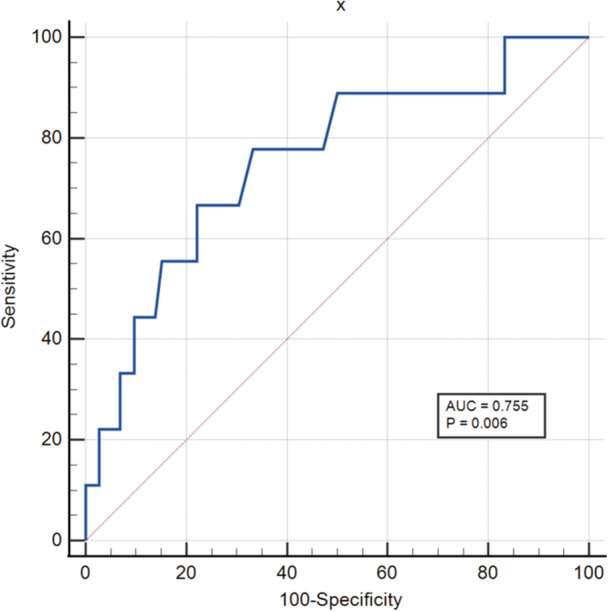
Optimal platelet cutoff value determined using receiver operating characteristics (ROC) analysis (AUC, area under the ROC curve).

In total, 10 SLE‐MAS patients (11.63%) died. All 86 patients were separated into two groups based on patient outcome (surviving or deceased) (Table 3). Univariate analysis revealed that the deceased group had lower platelet counts (43.69 ± 12.00 vs. 88.14 ± 8.937, *p* = .0112) and ferritin levels (1304 ± 246.8 vs. 61803 ± 30,813, *p* = .0245) than the surviving group. The proportion of patients with significantly elevated ferritin levels (>2000 ng/mL) was lower in the deceased group than in the surviving group (10.00% vs. 55.26%, *p* = .0071). According to previous studies, age has an influence on the prognosis of adult HLH. We performed a multiple logistic regression analysis using age, ferritin levels, and platelet counts to further evaluate the influence of these factors. The results revealed that age and platelet counts were independent factors associated with poor prognosis (Figure 1). A cutoff platelet count of ≤47 × 10^9^/L predicted a poor outcome. The discriminative ability of this prediction model was good (area under the ROC curve, 0.755; 95% CI, 0.647–0.844, *p* = .006) (Figure 2).

**Table 3:** Characteristics of SLE‐MAS in surviving and deceased cases.

	Deceased (*n* = 10)	Surviving (*n* = 76)	*p*
Age (mean ± SEM [range] years)	38.80 ± 6.441	30.21 ± 1.706	.1814
Male: female ratio	4/10 (40)	21/76 (27.63)	.4671
MAS as an initial manifestation of SLE, no./total no. (%)	5/10 (50)	39/76 (51.32)	>.9999
Coinfected, no./total no. (%)	1/10 (10)	7/76 (9.2)	>.9999
SLEDAI‐2000	15.00 ± 2.454	16.75 ± 1.026	.8078
Nephritis, no./total no. (%)	5/10 (50)	39/76 (51.32)	.588
Myositis, no./total no. (%)	0/10 (0)	8/76 (10.53)	.588
Cutaneous involvement, no./total no. (%)	4/10 (40)	49/76 (64.47)	.1727
Oral ulcer, no./total no. (%)	3/10 (30)	28/76 (36.84)	>.9999
Serositis, no./total no. (%)	4/10 (40)	21/76 (27.63)	.4671
Vasculitis, no./total no. (%)	1/10 (10)	9/76 (11.84)	>.9999
Arthritis, no./total no. (%)	2/10 (20)	28/76 (36.84)	.4828
Fever, no./total no. (%)	7/10 (70)	58/76 (76.32)	.7007
Hepatomegaly, no./total no. (%)	3/10 (30)	32/76 (42.11)	.5183
Splenomegaly, no./total no. (%)	3/10 (30)	37/76 (48.68)	.5141
Lymphadenopathy	3/10 (30)	27/76 (35.53)	.9282
WBC count, mean ± SEM (range) × 10^9^/L	4.123 ± 1.353	2.392 ± 0.2540	.0936
Hgb, mean ± SEM (range) g/L	91.65 ± 5.420	89.71 ± 2.365	.0936
Platelet count, mean ± SEM (range) × 10^9^/L	43.69 ± 12.00	88.14 ± 8.937	.0112
Complement C3 (g/L)	0.5230 ± 0.08780	0.4739 ± 0.04365	.4268
Complement C4 (g/L)	0.1102 ± 0.04827	0.09850 ± 0.01165	.5713
LDH, mean ± SEM (range) IU/L	2209 ± 629.3	1306 ± 120.4	.2131
Triglyceride (mg/dL)	278.4 ± 87.15	349.8 ± 30.04	.5355
(Ferritin > 2000 ng/mL)/(ferritin ≤2000 ng/mL) ratio	1/9 (10)	42/34 (55.26)	.0148
Ferritin, mean ± SEM (range) ng/mL	1304 ± 246.8	61,803 ± 30,813	.0245
Fibrinogen	250.3 ± 65.69	310.3 ± 29.91	.261
CRP, mean ± SEM (range) mg/L	48.94 ± 28.43	64.07 ± 22.58	.5599
ESR	55.17 ± 10.17	60.48 ± 4.783	.7961
Steroid pulse therapy, no./total no. (%)	10/10 (100)	63/76 (82.89)	.3475
IVIG, no./total no. (%)	3/10 (30)	34/76 (44.74)	.5044
Cyclosporine, no./total no. (%)	2/10 (20)	23/76 (30.26)	.7166
Supportive intensive care, no./total no. (%)	0/10 (0)	4/76 (5.26)	>.9999

Abbreviations: ALT, alanine aminotransferase; AST, aspartate aminotransferase; CRP, C‐reactive protein; ESR, erythrocyte sedimentation rate; Hgb, hemoglobin; IVIG, intravenous immunoglobulin; LDH, lactate dehydrogenase; MAS, macrophage activation syndrome; RNP, anti‐U1‐ribonucleoprotein; SEM, standard error of the mean; SLE, systemic lupus erythematosus; SLEDAI‐2000, Lupus Erythematosus Disease Activity Index 2000; WBC, white blood cell.

## 4 DISCUSSION

In total, 86 cases of SLE‐MAS were included in our retrospective analysis. We observed a female predominance in SLE‐MAS (29.07% of the patients were male), which is similar to previous studies on MAS in adults^2^, ^5^, ^15^ and juvenile SLE with MAS.^16^ Because male patients typically account for only 5%–9.6% of the SLE population,^17^, ^18^ the prevalence of MAS in male lupus patients was high. In agreement with previous data,^2^ fever, and cutaneous involvement were both cardinal features of adult hemophagocytic syndrome. In our study, 97.67% of the patients were administered a prednisone dose of greater than 100 mg per day, and 84.88% of the patients were administered a pulse of methylprednisolone. These observations are consistent with previous reports on therapeutic drug administration in SLE‐MAS patients.^2^, ^5^, ^15^ The mortality rate for patients with SLE‐MAS was 11.63%, which is similar to that reported in previous studies.^5^, ^15^ In contrast to previous studies,^3^ direct Coombs positive test results were not infrequent, at 15.12% in SLE‐MAS patients and 23.40% in patients with MAS as an initial manifestation of SLE.

Abdirakhmanova et al. found that the clinical manifestations of MAS can occur before a diagnosis of pediatric SLE is made.^19^ In our study, more than half of the SLE‐MAS patients presented with MAS as an initial manifestation of SLE, which is closer to previous reports.^15^ Because a marked improvement in the outcome of hemophagocytic syndrome can be attained when treating the underlying cause, it is crucial that SLE is identified as the underlying disease when hemophagocytic syndrome presents as an initial manifestation.

SLEDAI‐2K scores in SLE‐MAS patients were high, consistent with previous studies,^4^, ^5^, ^20^ and were higher in SLE patients with hemophagocytic syndrome as an initial manifestation (18.34 ± 1.240 vs. 14.45 ± 1.393, *p* = .03). Patients with MAS as an initial manifestation of SLE also demonstrated lower C3 levels, lower C4 levels, and a higher proportion of oral ulcers and serositis compared with patients with MAS that occurred during the course of lupus. Because these measures are all manifestations of lupus activity, we inferred that SLE disease activity was higher in SLE patients with MAS as an initial manifestation. Because the SLEDAI‐2K scores of patients without infections were as high as those in patients with infections, we inferred that infection may trigger hemophagocytic syndrome by inducing lupus activity and/or exacerbating lupus activity. In addition, only 26.74% of all lupus‐MAS patients in our study had infections. Thus, we conclude from our results that SLE disease activity was the predominant trigger for hemophagocytic syndrome, consistent with the research findings of Afia Aziz et al.^1^

Because SLE‐MAS patients typically show fever, it can be difficult to diagnose a coinfection in SLE‐MAS patients with only fever as a symptom. Nonetheless, because the treatment of SLE‐MAS patients with an infection differs markedly, it is crucial that a coinfection is diagnosed. A coinfection can burden SLE‐MAS patients with increased antigen presentation and T‐cell activation.^21^ Infections in SLE‐MAS patients are primarily diagnosed by bacterial culture, viral IgM antibody positive, or viral PCR positive. If MAS is caused by a bacterial or viral infection, treatment should focus on antibiotic or antiviral agents. If MAS is caused by lupus activity, sufficient hormones and immunosuppressants are needed to control lupus activity.

The overall mortality rate of patients with SLE‐MAS was 11.63% (10 out of 86 patients). The observed mortality rate was similar to the reported mortality rate of autoimmune‐MAS.^2^, ^5^, ^15^ Our results showed that mortality and thrombocytopenia are associated. This is consistent with the results of a previous report on all subtypes of secondary HLH by Li et al.^22^ Shen et al. also reported that the platelet count was an independent risk factor for HLH, with a platelet count of <58.5 × 10^9^/L acting as an optimal cutoff.^23^ According to our results, a platelet count of ≤47 × 10^9^/L was a predictor of a poor outcome.

Ferritin is regarded as the best‐discriminating biomarker in early secondary HLH^24^ and between MAS and non‐MAS in childhood‐onset SLE at diagnosis.^25^ Ferritin levels >662.5 ng/mL predict the occurrence of MAS in SLE.^15^ Debaugnies et al. reported that hyperferritinemia was a risk factor for poor prognosis.^26^ Our present study revealed that ferritin levels were lower in patients who eventually died than in the surviving group. However, ferritin was not found to be an independent factor associated with poor prognosis in multiple logistic regression analysis. Kumakura et al. also reported that SLE‐MAS was not associated with hyperferritinemia.^27^ Finally, we were unable to demonstrate that a coinfection or CRP levels of >50 mg/L were markers of poor prognosis in patients with SLE‐MAS.^27^

This retrospective review includes a considerable number of patients with SLE‐MAS in one study. To accomplish our study, we extracted detailed information on presentations, laboratory examinations, treatments, and outcomes from each case report where the full text was available. While our research provides key details about SLE‐MAS that should be helpful for rheumatologists, our study has several limitations. First, because the full text of some medical records could not be obtained or the data were incomplete, not all published medical records were included. Second, because these medical records did not originate from the same center, there may be a large degree of heterogeneity in the laboratory indicators. Third, we did not compare SLE‐MAS with SLE or with other types of hemophagocytic syndrome. Finally, due to the timing of data collection being set at the onset of MAS, cytopenia secondary to MAS was difficult to distinguish from cytopenia secondary to SLE.

In conclusion, we provide evidence that lupus disease activity is the predominant trigger in MAS, especially in cases where MAS is an initial feature of SLE. Our results also suggest that cases of SLE‐MAS with a coinfection should be carefully identified to provide appropriate treatment. Finally, thrombocytopenia is a predictor of poor prognosis in patients with SLE‐MAS.

## AUTHOR CONTRIBUTIONS

**Jingya Wang**: Conceptualization; data curation; formal analysis; funding acquisition; investigation; methodology; project administration; resources; software; supervision; validation; visualization; writing—original draft; writing—review and editing. **Wei Rong**: Data curation; formal analysis; investigation; methodology; resources; software. **Haotian Yan**: Data curation; investigation; resources; software.

## CONFLICT OF INTEREST STATEMENT

The authors declare no conflict of interest.

## Supporting information


**Supplementary Figure 1.** Geographical distribution of the included patients.

Supporting information.

## Data Availability

The data that support the findings of this study are available from the corresponding author upon reasonable request.
