# Supplementary figures and images for: Eighty‐six cases of clinical characteristics and outcomes of systemic lupus erythematosus‐associated macrophage activation syndrome: A meta‐analysis study
Source: Immun Inflamm Dis. 2024 Aug 7;12(8):e1364. doi: 10.1002/iid3.1364 (PMC11304897; doi:10.1002/iid3.1364)

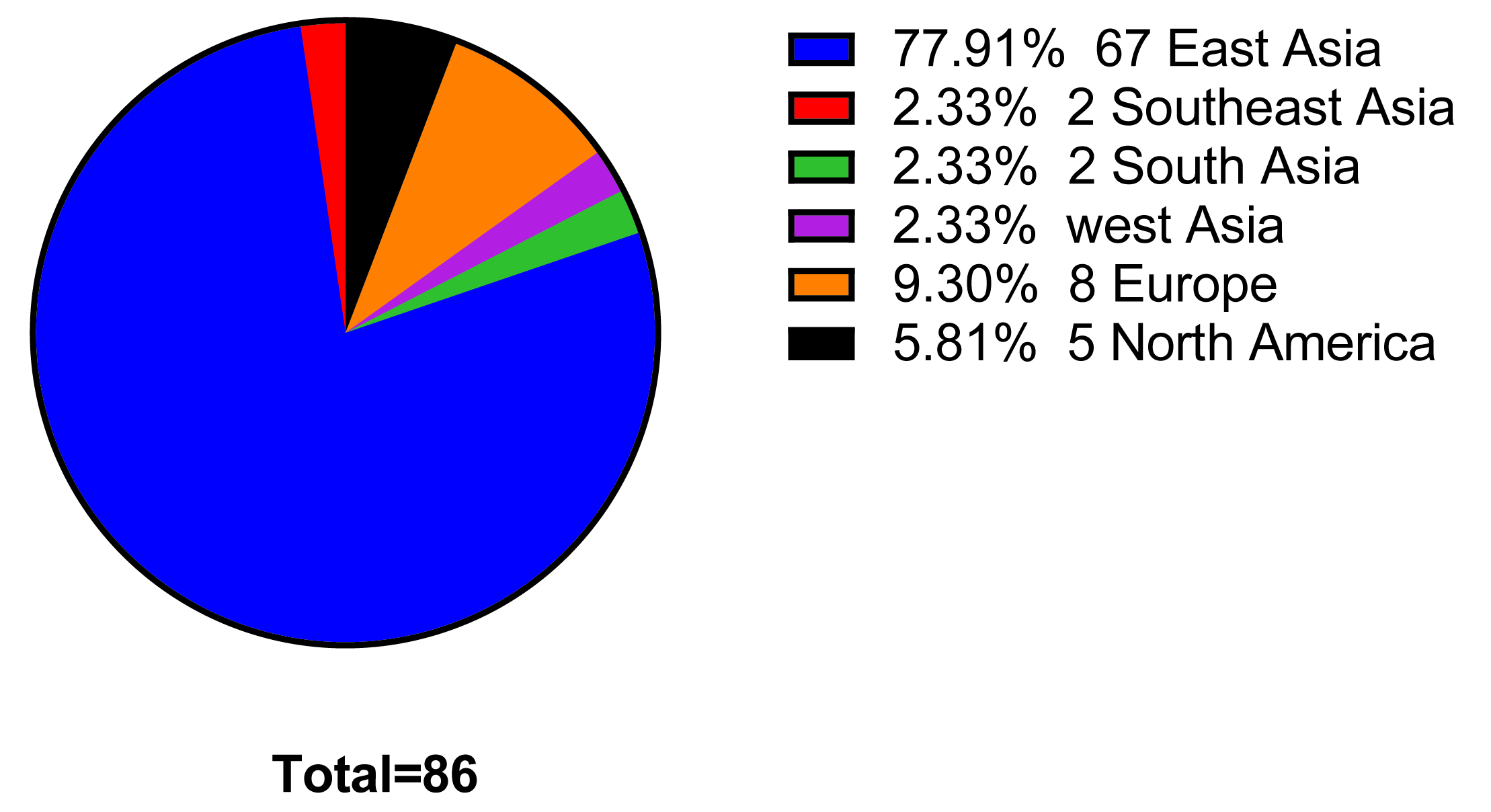

Supplement: Supplementary file 1 — Supplementary Figure 1. Geographical distribution of the included patients. [file IID3-12-e1364-s002.tif]
