# Supplementary material for: Eighty‐six cases of clinical characteristics and outcomes of systemic lupus erythematosus‐associated macrophage activation syndrome: A meta‐analysis study
Source: Immun Inflamm Dis. 2024 Aug 7;12(8):e1364. doi: 10.1002/iid3.1364 (PMC11304897; doi:10.1002/iid3.1364)
Supplement: Supplementary file 2 — Supporting information. [file IID3-12-e1364-s001.docx]

**Supplementary Table 1**. Flow chart of the included SLE-MAS patients.

**Identification**

**Screening**

**Included**

Records identified from:

Databases (n =686)

Registers (n =1)

Records removed before screening:

Duplicate records removed (n = 144)

Records marked as ineligible by automation tools (n = 31)

Records screened (n = 512)

Records were excluded (n = 359)

Reports sought for retrieval (n =153)

Reports not retrieved (n =19)

Reports assessed for eligibility (n =134)

Reports were excluded: Reason with incomplete data (n =58)

Reports of included studies (n =76)

98 cases meeting the diagnostic criteria were selected

12 cases were excluded because the full text could not be found or some data were missing

86 cases were included

| number | gender | age(years) | Pathogenic microorganism |
| --- | --- | --- | --- |
| 2 | M | 20 | methicillin-susceptible Staphylococcus aureus |
| 3 | M | 16 | E. coli |
| 6 | W | 27 | lung infection |
| 7 | W | 57 | Gram-negative coliforms |
| 11 | M | 48 | Staphylococcus aureus |
| 30 | W | 40 | Aspergillus fumigatus |
| 33 | M | 78 | methicillin-resistant staphylococcus |
| 54 | W | 15 | Mycobacterium avium |
| 64 | W | 48 | infection |
| 14-77 | W | 31 | infection |
| 15-78 | W | 50 | infection |
| 82 | W | 29 | Acinetobacter baumannii |
| 85 | W | 32 | Klebsiella acidogenes |
| 8 | W | 22 | BK virus |
| 14 | M | 12 | cytomegalovirus |
| 26 | W | 18 | Epstein Barr virus |
| 29 | M | 24 | Influenza virus B |
| 37 | W | 58 | cytomegalovirus |
| 41 | M | 25 | cytomegalovirus |
| 50 | W | 11 | Epstein Barr virus |
| 52 | W | 19 | Epstein Barr virus |
| 79 | W | 18 | Epstein Barr virus |
| 1-91 | W | 29 | herpes zoster |

**Supplementary Table 2**. Pathogenic microorganisms of CO infected persons

**Supplementary Table 3.** Characteristics of SLE-MAS with or without co-infection

|  | With infection  (n=23, 26.74%) | Without infection (n=63, 73.26%) | P |
| --- | --- | --- | --- |
| Age (mean± SEM (range) years) | 31.61 ± 3.628 | 31.06 ± 1.914 | 0.4005 |
| Cutaneous involvement, no. / total no. (%) | 10/23 (43.48) | 43/63 (68.25) | 0.0365* |
| Serositis, no. / total no. (%) | 9/23 (39.13) | 10/63 (15.87) | 0.0214* |
| Steroid pulse therapy, no. / total no. (%) | 18/23 (78.26) | 55/63 (87.30) | 0.0019* |

**Supplementary Figure 1**. Geographical distribution of the included patients.
